# The *MAB_3513c* gene plays a key role in intrinsic resistance of *Mycobacterium abscessus* to isoniazid and ethionamide

**DOI:** 10.1128/spectrum.02889-25

**Published:** 2025-11-28

**Authors:** Shuai Wang, Xiaofan Zhang, Xiange Fang, H. M. Adnan Hameed, Abdul Malik, Lihua Long, Yamin Gao, Cuiting Fang, Xirong Tian, Jinxing Hu, Xingyue Wang, Liqiang Feng, Tianyu Zhang

**Affiliations:** 1 State Key Laboratory of Respiratory Disease, Guangzhou Institutes of Biomedicine and Health, Chinese Academy of Sciences 74627, Guangzhou, China; 2 University of Chinese Academy of Sciences 74519, Beijing, China; 3 China-New Zealand Joint Laboratory on Biomedicine and Health, Guangzhou Institutes of Biomedicine and Health, Chinese Academy of Sciences 53042 , Guangzhou, China; 4 Department of Clinical Laboratory, Sun Yat-sen Memorial Hospital of Sun Yat-sen University 56713 , Guangzhou, China; 5 State Key Laboratory of Respiratory Disease, Guangzhou Medical University 26468, Guangzhou, China; 6 State Key Laboratory of Respiratory Disease, Guangzhou Chest Hospital, Guangzhou, China; 7 Guangzhou National Laboratory 612039, Guangzhou, China; MultiCare Health System, Tacoma, Washington, USA

**Keywords:** *Mycobacterium abscessus*, isoniazid, ethionamide, intrinsic resistance, *nudC*

## Abstract

Isoniazid (INH) and ethionamide (ETH) are important antitubercular analogs that inhibit mycolic acid synthesis. However, *Mycobacterium abscessus* (*M. abscessus*) exhibits intrinsic resistance to both drugs. The molecular mechanisms underlying this resistance remain poorly understood. We identified *MAB_3513*c (*nudC*), encoding a functional NADH pyrophosphatase NudC that hydrolyzes drug-NAD adducts, as being crucial for intrinsic resistance to INH and ETH in *M. abscessus*. Susceptibility to both antibiotics was markedly enhanced upon either deleting *nudC* or introducing the P226Q point mutation in this conserved residue. High-performance liquid chromatography–mass spectrometry analysis further demonstrated that the deletion of *nudC* resulted in accumulation of the ETH-NAD adduct and a decrease in its hydrolysis rate in ETH-treated *M. abscessus*. Size-exclusion chromatography analysis revealed that wild-type NudC predominantly forms dimers, whereas the P226Q mutant exists mainly as monomers, consistent with a loss of enzymatic function. A pivotal proline at position 226 governs its dimerization and enzymatic activity. To complement the *nudC* knockout *M. abscessus* strain, we introduced seven different versions of the gene separately, each producing a protein with a point mutation at different key residues: D133A, N148D, F156A, F193A, E207Q, E211Q, and R231Q. None of them restore the resistance to INH and ETH, illustrating that these sites are also critical for the catalytic activity of NudC. This discovery elucidates a previously unrecognized mechanism of drug resistance in *M. abscessus* and highlights NudC as a potential target for therapeutic intervention.

**IMPORTANCE:**

*Mycobacterium abscessus* poses a serious therapeutic challenge due to its extensive drug resistance. This study elucidates the molecular mechanism behind its intrinsic resistance to the critical antitubercular drugs isoniazid and ethionamide. We identify the NudC (MAB_3513c) enzyme as a central resistance factor that functions by hydrolyzing the active drug-NAD adducts. Crucially, we demonstrate that NudC’s enzymatic activity, dependent on its dimerization and key catalytic residues, is essential for this resistance mechanism. This finding establishes NudC as a promising therapeutic target for combating this highly drug-resistant pathogen.

## INTRODUCTION

*Mycobacterium abscessus* is an emerging opportunistic pathogen that causes a spectrum of human diseases, notably chronic pulmonary conditions and extrapulmonary infections affecting the skin, soft tissues, and central nervous system (1). It is the most frequently isolated rapidly growing nontuberculous mycobacterium among patients with structural lung diseases, such as bronchiectasis, cystic fibrosis, and chronic obstructive pulmonary disease (2, 3). Standard antibacterial therapy is often ineffective against these infections because they possess intrinsic high-level resistance to most antibiotics, including front-line anti-tuberculosis drugs isoniazid (INH) and the structurally related second-line drug ethionamide (ETH) (4, 5). A multidrug regimen containing an oral macrolide (clarithromycin or azithromycin) and intravenous aminoglycoside (amikacin), occasionally with a second-line antibiotic (e.g., β-lactams, quinolones, tetracyclines, or oxazolidinones), is recommended for the treatment of *M. abscessus* infections (6). However, treatment outcomes are frequently suboptimal, particularly for patients with chronic pulmonary infections and refractory infections, which often leads to relapse and death (7).

Intrinsic resistance of *M. abscessus* to antibiotics is mediated by multiple factors including the low permeability of the cell envelope, the expression of a wide range of antibiotic-target-modifying and antibiotic-modifying enzymes, and the induction of efflux pumps (4, 8). For instance, a recent study identified *embC*—a gene encoding an arabinosyltransferase involved in lipoarabinomannan biosynthesis—as a key determinant of cell envelope permeability and, consequently, intrinsic resistance in *M. abscessus* (9). Moreover, a functional *erm*(41) gene encodes an Erm methylase that modifies the 23S rRNA, thereby inducing resistance to macrolides and leading to treatment failure in certain patients (10). A comprehensive understanding of the genetic basis of intrinsic antibiotic resistance is a prerequisite for developing synergistic drug combinations, where one agent makes a failing antibiotic active by interfering with molecular determinants of intrinsic resistance. For example, when combined with β-lactam antibiotics, the β-lactamase inhibitor clavulanic acid prevents the degradation of these antibiotics, thereby maintaining their therapeutic efficacy (11, 12).

INH and ETH are prodrugs that upon activation inhibit the synthesis of mycolic acids, leading to *Mycobacterium tuberculosis* death. INH is activated by the catalase-peroxidase KatG to form the INH-NAD adduct, which inhibits the synthesis of essential mycolic acids by targeting the enoyl-acyl carrier protein reductase InhA (13). ETH, a structural analog of INH, is activated by the monooxygenase EthA to form the ETH-NAD adduct, which binds to InhA and inhibits mycolic acid biosynthesis (14, 15). However, *M. abscessus* exhibits intrinsic resistance to both drugs. Reportedly, resistance to INH in *M. abscessus* is linked to a loss of catalase-peroxidase function in the KatG enzyme (16). The introduction of the *katG* gene from *M. tuberculosis* into *M. abscessus* enhances its susceptibility to INH (17), yet the recombinant *M. abscessus* remains more resistant to INH than *M. tuberculosis*. This implies that intrinsic resistance of *M. abscessus* to INH arises not only from impaired KatG-dependent activation but also from other as-yet-unidentified factors.

In this study, we identified *MAB_3513*c (*nudC*), which encodes a functional NADH pyrophosphatase NudC that hydrolyzes the drug-NAD adducts, as a critical genetic determinant of intrinsic resistance to both INH and ETH in *M. abscessus*. Through gene editing and complementation of point mutated *nudC* in *nudC* knockout *M. abscessus* strain (ΔnudC), we demonstrated that the proline residue at position 226 and other seven key amino acid active sites in NudC are essential for conferring resistance.

## RESULTS

### *MAB_3513c* encodes a putative NADH pyrophosphatase (NudC)

A previous transposon sequencing study showed a significant depletion of transposon insertions in the *MAB_3513*c gene when the transposon mutant library was cultured in the presence of sub-inhibitory concentrations of ETH, compared to drug-free conditions (18). This finding suggests that *MAB_3513*c may contribute to ETH resistance. The *MAB_3513*c was identified as a *nudC* gene, encoding a putative protein with 60.44% identity to *M. tuberculosis* NudC and 60.75% identity to *Mycobacterium bovis* BCG NudC using BLASTp (National Center for Biotechnology Information [NCBI]) (Fig. S1). NudC is known to catalyze the hydrolysis of NADH to adenosine monophosphate (AMP) and nicotinamide mononucleotide (NMNH) (19). Previous studies have shown that NudC^BCG^ degrades the active forms of INH (INH-NAD) and ETH (ETH-NAD). Specifically, incubation of INH-NAD or ETH-NAD with NudC^BCG^ results in the release of AMP together with INH-NMNH or AMP and ETH-NMNH, respectively (20). Moreover, *nudC*^BCG^ inactivation leads to increased susceptibility to these drugs in BCG (20).

### Deletion of *nudC* increases *M. abscessus* susceptibility to ETH and INH

**Fig 1:**
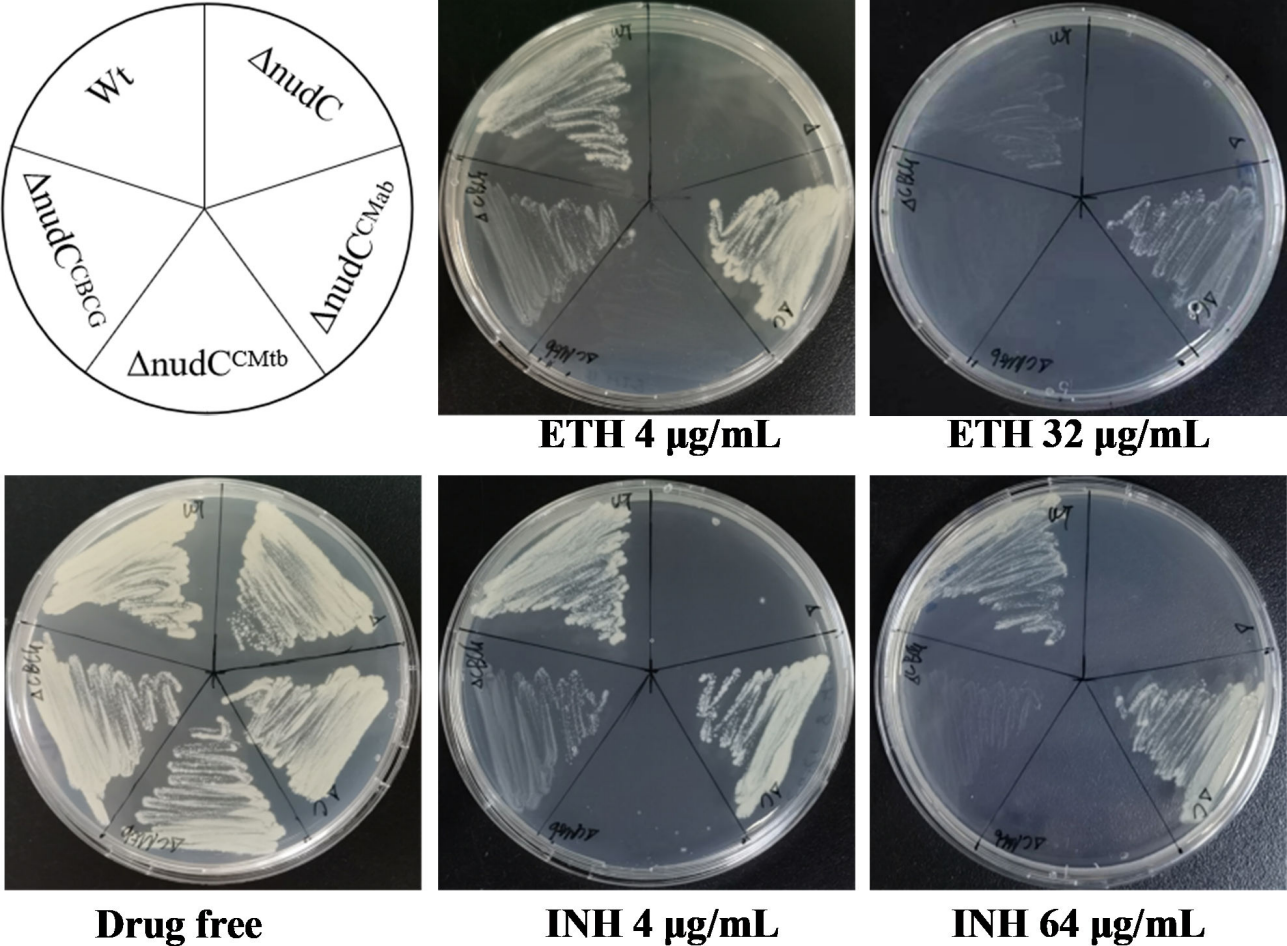
Susceptibilities of different *M. abscessus* strains to INH and ETH on 7H11 agar. The bacterial suspension was spread on the 7H11 containing different antibiotics. The plates were incubated for 3 days. Wt: wild-type *M. abscessus*; ΔnudC: *M. abscessus nudC* deletion mutant; ΔnudC^CMab^: ΔnudC complemented with *M. abscessus nudC*; ΔnudC^CBCG^: ΔnudC complemented with BCG *nudC*; ΔnudC^CMtb^: ΔnudC complemented with *nudC^Mtb^*.

Based on the above analysis, we hypothesize that *nudC* encodes a functional NudC in *M. abscessus* that contributes to resistance by a mechanism similar to that found in BCG (20). To validate this, we generated a selectable marker-free, isogenic *nudC* deletion mutant (ΔnudC) using CRISPR-assisted recombineering (21) (Fig. S2). A complemented strain (ΔnudC^CMab^) was constructed by reintroducing *M. abscessus nudC* under the control of the strong mycobacterial *hsp60* promoter via an integrative plasmid. Drug susceptibility testing (DST) demonstrated that ΔnudC was markedly more susceptible to ETH than wild-type (Wt) *M. abscessus* (Table 1; Fig. 1). Complementation restored the susceptibility to Wt levels. Given that INH shares a similar mechanism of action with ETH, we further assessed the susceptibility of ΔnudC to INH and five other antibiotics with distinct mechanisms of action: amikacin, rifabutin, levofloxacin, clarithromycin, and azithromycin. The results showed that ΔnudC exhibited dramatically increased susceptibility to INH, with a minimal inhibitory concentration (MIC) less than 1/128 of that to Wt (Table 1; Fig. 1). However, no significant changes in susceptibility were observed for amikacin, rifabutin, levofloxacin, clarithromycin, or azithromycin. Additionally, deletion of *nudC* had minimal impact on bacterial growth (Fig. S3), indicating that *nudC* specifically influences susceptibility to INH and ETH without affecting overall fitness.

**TABLE 1:** MICs of various drugs against different *M. abscessus* strains^*a*^^,^^*b*^

Strains	Antibiotics/ MIC (μg/mL)						
	INH	ETH	Rifabutin	Amikacin	Levofloxacin	Clarithromycin^*c*^	Azithromycin
Wt	>128	>128	2	8	32	4, 32, 64	32
ΔnudC	1	16	2	8	32	4, 32, 64	32
ΔnudC^CMab^	>128	>128	2	8	32	4, 32, 64	32
ΔnudC^CBCG^	>128	128	2	8	32	8, 32, 128	32
ΔnudC^CMtb^	1	16	2	8	32	8, 32, 128	32
P226Q	1	16	2	8	32	4, 32, 64	32
P226Q^CQ^	1	16	2	8	32	8, 32, 128	64
P226Q^CMab^	>128	>128	2	8	32	8, 32, 128	64
ΔnudC^CD133A^	1	8	2	8	32	8, 32, 128	32
ΔnudC^CN148D^	1	16	2	8	32	8, 32, 128	32
ΔnudC*^C^*^F156A^	1	16	2	8	32	8, 32, 128	64
ΔnudC^CF193A^	1	16	2	8	32	8, 32, 128	64
ΔnudC^CE207Q^	1	16	2	8	32	8, 32, 128	32
ΔnudC^CE211Q^	1	16	2	8	32	8, 32, 128	32
ΔnudC^CR231Q^	1	16	2	8	32	8, 32, 128	64

^*a*^ Wt: wild-type *M. abscessus*; ΔnudC: *M. abscessus nudC* deletion mutant; ΔnudC^CMab^: ΔnudC complemented with *M. abscessus nudC*; ΔnudC^CBCG^: ΔnudC complemented with *nudC*^BCG^; ΔnudC^CMtb^: ΔnudC complemented with *nudC^Mtb^*; P226Q: *M. abscessus* carrying a *nudC *encoding a P226Q point mutation; P226Q^CQ^: the P226Q strain containing an extra *nudC* encoding a P226Q point mutation; P226Q^CMab^: the P226Q strain containing an extra* M. abscessus nudC*; ΔnudC^CD133A^, ΔnudC^CN148D^, ΔnudC^CF156A^, ΔnudC^CF193A^, ΔnudC^CE207Q^, ΔnudC^CE211Q^, and ΔnudC^CR231Q^: ΔnudC strain complemented with a *nudC* encoding a D133A, N148D, F156A, F193A, R231Q, E207Q, or E211Q point mutation*, *respectively*.*

^*b*^ Broth microdilution method was used to determine the MICs.

^*c*^ The MICs of clarithromycin against different *M. abscessus* strains were observed in 3, 7, and 14 days, respectively. The MIC was defined as the lowest drug concentration that prevented visible bacterial growth. The experiment was performed in triplicate and repeated three times.

### The *nudC* confers resistance to INH and ETH by encoding NudC that hydrolyzes drug-NAD adducts effectively

**Fig 2:**
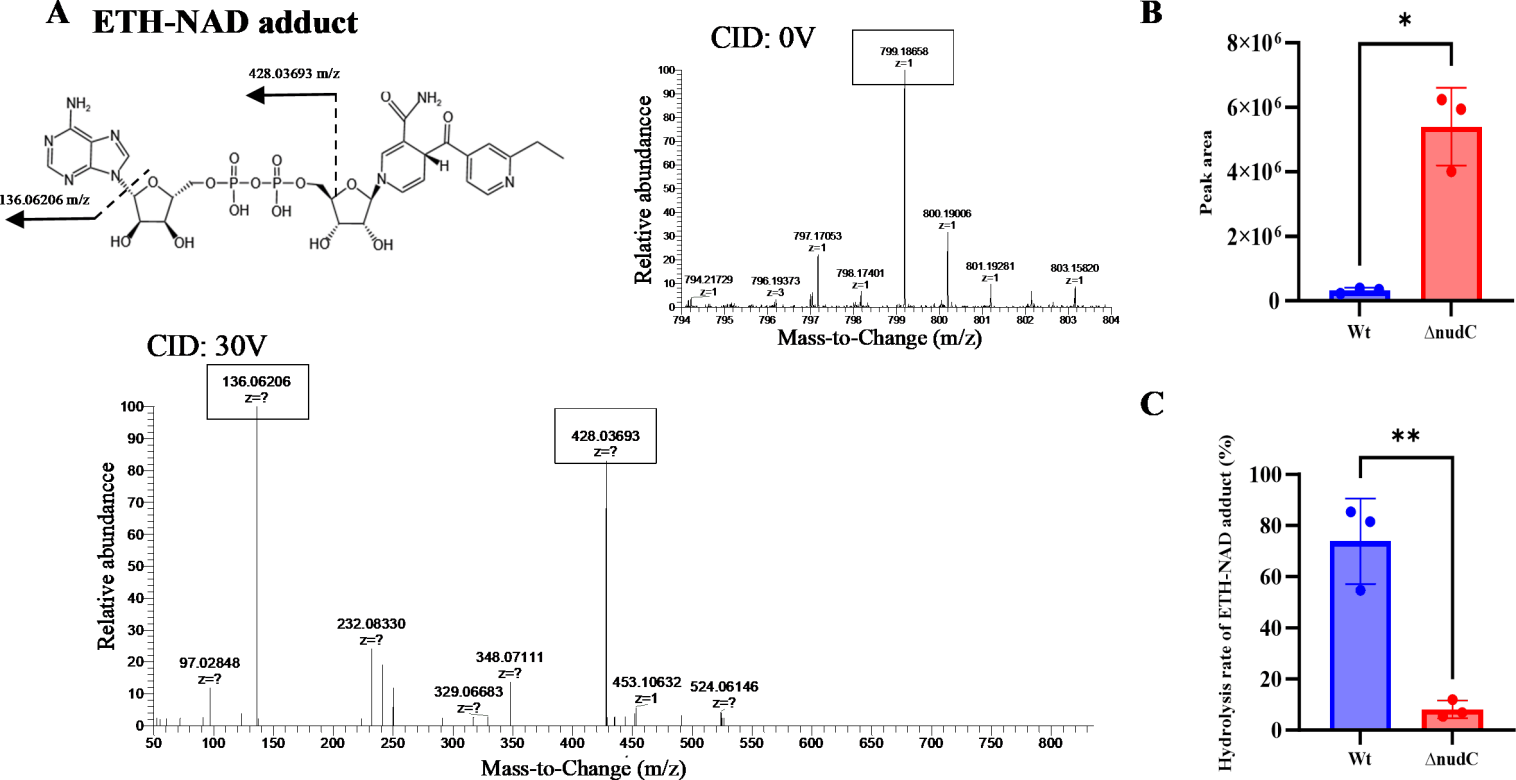
Inactivation of NudC results in accumulation of ETH-NAD adduct and decrease of its hydrolysis rate in ETH treated *M. abscessus*. (**A**) The structures and MS/MS fragmentation spectra provide confirmatory evidence of mass matching to the predicted ETH-NAD adduct and show fragmentations consistent with adenine and adenosine diphosphate moieties of the adduct. (**B**) Quantification of the ETH-NAD adduct in the Wt and ΔnudC strains. (**C**) Hydrolysis rates of the ETH-NAD adduct in Wt and ΔnudC. ^*^P < 0.05, ^**^P < 0.01. Data are means ± SD of three cultures.

To further substantiate the hypothesis that *nudC* encoding a highly active NudC is capable of hydrolyzing drug-NAD adducts, we took ETH-NAD adduct as an example and measured the impact of deleting *nudC* on the accumulation of ETH-NAD adduct in ETH-treated *M. abscessus*. We first exposed *M. abscessus* to ETH and prepared extracts for analysis by high-performance liquid chromatography–mass spectrometry (HPLC/MS). The observed molecule ([M + H] = 799.18658) matched the predicted mass of the ETH-NAD adduct (22, 23). Its high-resolution fragmentation pattern specifically included two major mass ions ([M + H] = 136.06206 and [M + H] = 428.03693) corresponding to the adenine and adenosine diphosphate moieties of the ETH-NAD adduct (22), respectively (Fig. 2A). The intensity of adduct in ΔnudC is accumulating approximately 13-fold higher than that in Wt (Fig. 2B). The intensity of ETH-NMNH was also detected to further verify the role of *nudC*. The ratio of ETH-NAD adduct hydrolyzed into ETH-NMNH in ΔnudC decreased to about lower than 1/9 of that in Wt (Fig. 2C). Collectively, these findings demonstrated that *nudC* encodes a functional NudC that hydrolyzes ETH-NAD adduct and may also hydrolyze INH-NAD adduct, thereby contributing to the intrinsic resistance of *M. abscessus* to ETH and INH.

### A single amino acid substitution (P226Q) in NudC^Mab^ modulates resistance to INH and ETH

Multiple sequence alignment revealed that NudC is highly conserved among mycobacterial species, with a notable divergence at residue 226 (*M. abscessus* numbering). INH- and ETH-sensitive *M. tuberculosis* encodes a glutamine (Q226) at this position, while intrinsic resistant species such as *M. abscessus*, BCG, and *Mycobacterium smegmatis* harbor a proline (P226) (Fig. S1). A previous study suggested that this residue is located at the dimerization interface of NudC, where glutamine disrupts dimer formation, while proline stabilizes it and is associated with resistance to INH and ETH (20). To assess the functional significance of this residue, we first expressed NudC^Mtb^ in the ΔnudC strain. The heterologous protein failed to restore resistance to INH or ETH (Fig. 1; Table 1). However, heterologous expression of *nudC*^BCG^ partially restored resistance to INH and ETH, although not to the same level as the *nudC*^Mab^ (Fig. 1; Table 1). This may be due to lower enzymatic activity of NudC^BCG^ compared to NudC^Mab^.

**Fig 3:**
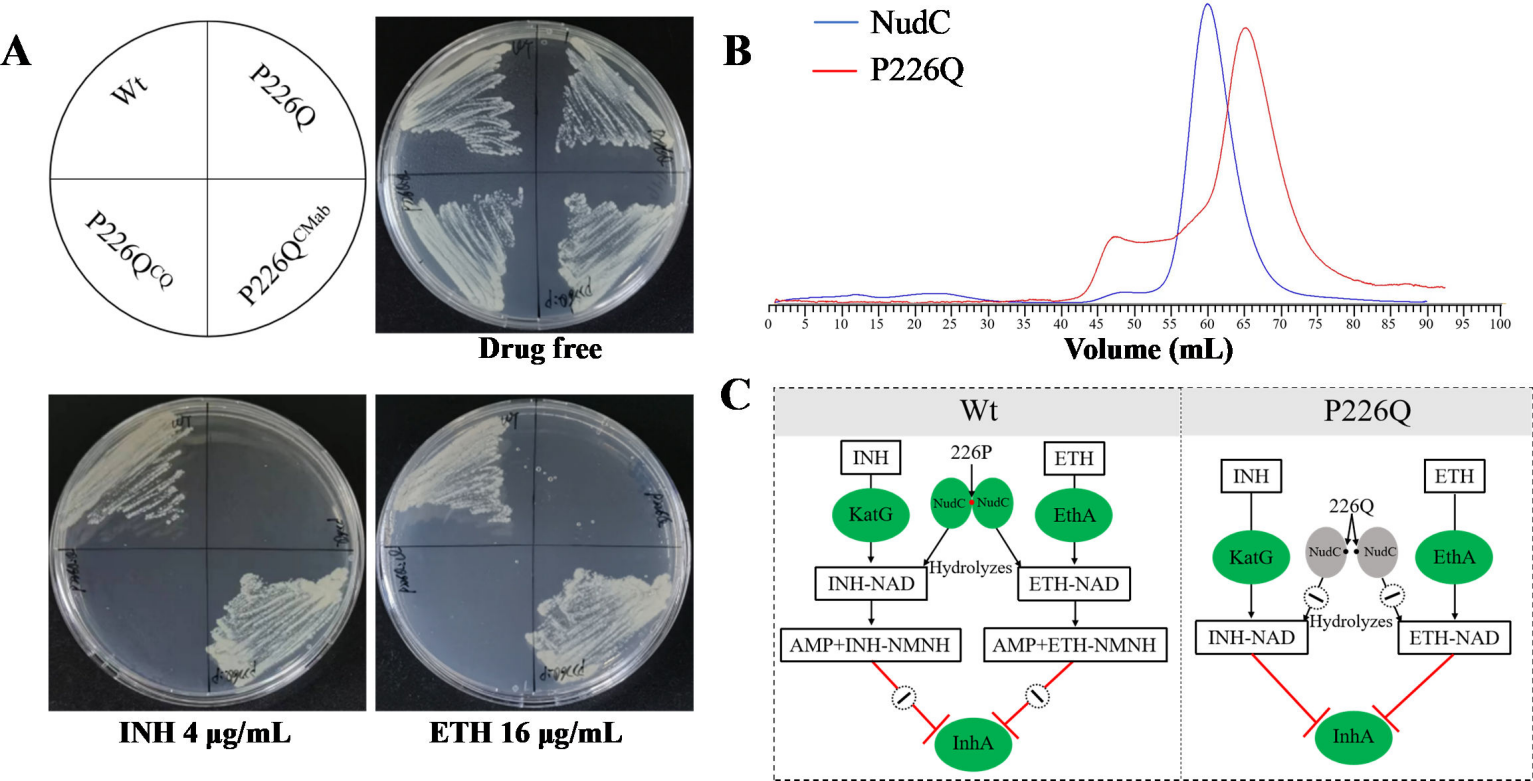
The amino acid at the 226th position in NudC is crucial for mediating *M. abscessus*’ resistance against both INH and ETH. (**A**) DST of various *M. abscessus* strains to INH and ETH. Bacterial suspensions were plated on 7H11 agar supplemented with the indicated antibiotics and incubated for 3 days. (**B**) Size-exclusion chromatography analysis of purified NudC and its mutant NudC^P226Q^. (**C**) Proposed model of intrinsic resistance to INH and ETH in *M. abscessus*. In *M*. *abscessus*, the presence of a proline (P) at position 226 promotes dimerization, allowing NudC to hydrolyze INH-NAD and ETH-NAD, which confers intrinsic resistance to both INH and ETH. Conversely, the substitution of this proline with a glutamine (Q) in NudC prevents dimer formation. This inhibition fails to degrade the active INH-NAD and ETH-NAD adduct, making the bacteria sensitive to these antibiotics. Wt: wild-type *M. abscessus*; P226Q: *M. abscessus* carrying a *nudC* encoding a P226Q point mutation; P226Q^CQ^: P226Q strain carrying an additional *nudC* encoding a P226Q point mutation; P226Q^CMab^: P226Q strain carrying a wild-type *M. abscessus nudC*.

Next, to directly evaluate the role of the NudC P226 residue in *M. abscessus*, we generated a point mutant strain (P226Q) by substituting proline with glutamine at this position. DST demonstrated that P226Q exhibited markedly increased sensitivity to both INH and ETH, phenocopying the ΔnudC strain (Table 1; Fig. 3A). Moreover, complementation with *M. abscessus nudC* restored resistance in P226Q (P226Q^CMab^), whereas complementation with the mutant *nudC*^P226Q^ (P226Q^CQ^) failed to restore resistance. To explore the structural basis of this resistance phenotype, we performed size-exclusion chromatography on purified NudC and NudC^P226Q^ proteins. Wt NudC eluted earlier than the mutant protein, consistent with a dimeric conformation for NudC, while a monomeric for NudC^P226Q^ (Fig. 3B). Together, these results demonstrated that a single amino acid substitution at position 226 critically governs the intrinsic resistance of *M. abscessus* to INH and ETH, likely by modulating NudC dimerization. These findings highlight how species-specific structural variation in drug-modifying enzymes can influence susceptibility to key antimicrobials.

### Identification of seven key active sites in NudC with DST

A structural study revealed that *M. abscessus* NudC contains a unique tower domain and the residue P226 within a conserved sequence (QPWPFPxS) is critical for the enzymatic activity of NudC (24). The seven key amino acid active sites (D133, N148, F156, F193, E207, E211, and R231) were identified from the structural and enzymatic activity analysis of NudC (24). However, it remained unknown whether the strains harboring these point mutations are sensitive to INH and ETH; thus, we investigated them in our current study. After obtaining the complemented strains ΔnudC^CD133A^, ΔnudC^CN148D^, ΔnudC^CF156A^, ΔnudC^CF193A^, ΔnudC^CE207Q^, ΔnudC^CE211Q^, and ΔnudC^CR231Q^ expressing the corresponding point mutations of *nudC*, respectively, the MICs of INH and ETH against them were detected. The complementation of ΔnudC with point-mutated *nudC* did not restore resistance to INH and ETH compared to Wt (Table 1). The MICs were basically consistent with that against ΔnudC. The agar DST was conducted to validate the MIC results (Fig. S4). These findings suggested that the role of point mutation in *nudC* aligns with the knockout of *nudC*. Consequently, these seven amino acid sites are imperative for the optimal functionality of NudC.

## DISCUSSION

Treatment of *M. abscessus* infections remains highly challenging due to its intrinsic resistance to a broad spectrum of antibiotics, resulting in poor therapeutic outcomes. Therefore, a deeper understanding of the intrinsic resistance mechanisms of this pathogen, in combination with drug development and repurposing efforts, is necessary for improving the therapeutic success of *M. abscessus* infections. This study demonstrates that the intrinsic resistance of *M. abscessus* to INH and ETH is mediated by the hydrolysis of the INH-NAD and ETH-NAD adduct by NudC (Fig. 3C). The *M. abscessus* complex can be classified into three subspecies based on the whole-genome sequencing: *M. abscessus* subsp. *abscessus*, *M. abscessus* subsp. *massiliense*, and *M. abscessus* subsp. *bolletii* (25). But the sequences of NudC among different subspecies of *M. abscessus* are completely identical (Fig. S5). Therefore, we did not repeat the MIC testing across subspecies. The previously reported structures of mycobacteria NudC (PDB code: 8ZB3) (24) and *Escherichia coli* NudC (PDB code: 2GB5) (19) are highly similar, suggesting that they share similar mechanisms to hydrolyze NAD. However, mycobacterial NudC contains a unique tower domain and a distinctive arginine residue (R231) within the conserved QPWPFPxS motif, while *E. coli* NudC contains a well-defined zinc-binding motif. The residue P226 of NudC locates on the dimer interface of the C-terminal domain, which is the crucial determinant for maintaining its dimeric structure and plays a key role in INH and ETH intrinsic resistance in *M. abscessus* (Fig. 3C). DST was conducted on the point mutant corresponding to the seven key amino acid active sites of NudC respectively, and the results were consistent with the enzymatic activity of these mutants reported in another study (24). These key amino acid sites contribute to the stability of the tower domain and the catalytic activity of NudC (24).

Previous studies have established that InhA is the primary target of INH and ETH in mycobacteria, which is inhibited by a covalent INH-NAD adduct (13, 26, 27) or ENH-NAD adduct (14, 15). The two drug-NAD adduct ultimately results in the inhibition of mycolic acid biosynthesis and the death of mycobacteria cells by blocking the InhA. Sequence alignment of InhAs from *M. abscessus* and *M. tuberculosis* shows 88.1% identity (Fig. S6), indicating highly conserved functions. Key active site residues interacting with the INH-NAD adduct (S94, F149, M155, Y158, G192, P193, L218, and W222) (13) are identical in both species (Fig. S6). Notably, InhA mutations commonly found in INH- and ETH-resistant *M. tuberculosis* clinical isolates (S94A, I21T, and I95P) (28) were absent in *M. abscessus* (Fig. S6). Taken together, we speculate that InhA^Mab^ is also the target of INH and ETH like InhA^Mtb^. The HPLC/MS results further demonstrate that NudC could hydrolyze the ETH-NAD adduct, the deletion of *nudC* resulting in accumulation of ETH-NAD adduct and decrease of its hydrolysis rate in ETH-treated *M. abscessus*. Therefore, *M. abscessus* resistance to INH and ETH mainly appears to be NudC-mediated hydrolysis of drug-NAD adduct. Our findings enhance the understanding of intrinsic resistance to INH and ETH in *M. abscessus*.

Although deletion of *nudC* in *M. abscessus* significantly increased susceptibility to both INH and ETH, the resulting MICs of these drugs to *M. abscessus* remained considerably higher than those observed in INH- and ETH-sensitive *M. tuberculosis* strains. This suggests that additional mechanisms beyond NudC contribute to the intrinsic resistance of *M. abscessus* to these antibiotics. One key factor influencing INH susceptibility across mycobacterial species is the activity of KatG, a catalase-peroxidase that activates INH (16). Notably, heterologous expression of KatG from *M. tuberculosis* in *M. abscessus* significantly enhances its sensitivity to INH, underscoring the importance of INH activation efficiency in *M. abscessus*’ resistance (17). Likewise, in *Mycobacterium marinum*, which is intrinsically resistant to INH and ETH, the introduction of *M. tuberculosis katG* or *ethA* genes renders *M. marinum* more susceptible to INH or ETH, respectively (29), illustrating the impact of varying prodrug activation on mycobacterial drug resistance. Beyond activation enzymes, regulatory pathways also influence ETH susceptibility. The transcriptional regulator MarR, which negatively regulates *mmpS5-mmpL5*, has been implicated in ETH resistance, and its deletion enhances ETH sensitivity in *M. abscessus* (18). NudC removes the NAD^+^ cap from the 5′ terminus of prokaryotic mRNA through pyrophosphohydrolysis, thereby destabilizing the transcript and promoting its degradation (19). This process highlights NudC’s crucial role in RNA metabolism and homeostasis. Unlike *M. tuberculosis*, where NudC exists as monomers, in most mycobacteria, including *M. abscessus*, NudC forms as functional dimers, suggesting species-specific variations in structural or regulatory mechanisms. Attempts to restore NudC function in a knockout strain via a multicopy plasmid were unsuccessful (data not shown), whereas complementation with a single-copy integrative system succeeded, implying that *nudC* overexpression might disturb cellular homeostasis in *M. abscessus*. These observations accentuate NudC’s significance in bacterial physiology and call for further inquiry into its necessity across mycobacterial species and the physiological consequences of its expression levels.

In summary, this study identifies the gene *MAB_3513c*, which encodes the NudC responsible for hydrolyzing drug-NAD adducts, as a key determinant of *M. abscessus*’ intrinsic resistance to INH and ETH. We demonstrate that a variation at amino acid position 226, where *M. abscessus* has proline instead of glutamine found in *M. tuberculosis*, promotes NudC dimer formation, consequently enabling *M. abscessus*’ resistance. Given this crucial role, NudC emerges as a potential therapeutic target, and its inhibition could enhance the efficacy of INH or ETH against *M. abscessus* infections. Our findings provide new insights into the molecular basis of *M. abscessus* resistance and lay the groundwork for future therapeutic strategies.

## MATERIALS AND METHODS

### Strains and culture conditions

*E. coli* DH5α was cultured in Luria-Bertani (LB) broth or on LB agar plates at 37°C. *M. abscessus* subsp. *abscessus* GZ002 (NCBI GenBank accession numbers CP034181), a previously described clinical isolate (30), was cultured at 37°C in Middlebrook 7H9 broth (Difco) supplemented with 10% oleic acid-albumin-dextrose-catalase (OADC; Difco) and 0.05% Tween 80, or on Middlebrook 7H11 agar (Difco) containing 10% OADC. Where required, kanamycin (100  µg/mL) and zeocin (30  µg/mL) were added to the media for plasmid selection and maintenance.

### DST

The MICs of different *M. abscessus* strains were determined using the broth microdilution method in 7H9 medium. Bacterial cultures in the exponential growth phase were adjusted to an inoculum of 5 × 10^6^ CFU/mL. A volume of 150 µL of bacterial suspension was added to each well of a 96-well plate, except for the first column, which received 297 µL. To the first well of the first column, 3 µL of the antibiotic stock solution was added, followed by twofold serial dilutions. Plates were incubated at 37°C for 3, 7, and 14 days for clarithromycin and 3 days for other drugs. MICs were determined by visual inspection of bacterial growth.

For solid agar-based DST, bacterial cultures were adjusted to an OD_600_ of 0.1 and streaked on the 7H11 agar plates (with or without antibiotics) using a sterile inoculation loop. When ΔnudC and its complemented strains reached an OD_600_ of 0.6 to 0.8, the cultures were diluted in a 10-fold series up to 10^−5^ with 7H9 medium and spotted on the 7H11 plates, either with or without antibiotics. Plates were incubated at 37°C for 3 days, and colony growth was monitored and photographed.

### Construction of *nudC* knockout and point mutation strains

The *nudC* deletion and base-editing mutants were generated via CRISPR-associated recombineering as previously described (21). Briefly, double-stranded donor DNA fragments carrying either an in-frame deletion or a point mutation resulting in a P226Q substitution were co-electroporated with a crRNA plasmid targeting *nudC* into *M. abscessus* containing pJV53-Cpf1 plasmid (21). Recombinant clones were selected for successful allelic exchange via a double-crossover event using 30 µg/mL zeocin, 100 µg/mL kanamycin, and 30 ng/mL anhydrotetracycline. Recombinant colonies were screened for successful allelic exchange by PCR and confirmed by Sanger sequencing. The confirmed strains were designated as ΔnudC (*nudC* knockout) and P226Q (point mutant) strains, respectively.

### Construction of complemented strains

To functionally complement the ΔnudC mutant, a full-length homolog of *nudC* under the control of the *hsp60* promoter was cloned into pRH2502 (an integrative plasmid vector) and introduced into the deletion mutant. We constructed plasmids carrying point mutations corresponding to seven active-site residues (D133A, N148D, F156A, F193A, E207Q, E211Q, or R231Q) of *nudC* on the pRH2502 backbone and electroporated them into Δ*nudC*, respectively. Successful integration and expression of the complemented genes were confirmed by antibiotic selection, PCR, and Sanger sequencing.

### Bacterial growth assay

Growth kinetics of Wt, ΔnudC, and the complemented strain (ΔnudC^CMab^) were evaluated to assess the impact of *nudC* deletion on bacterial proliferation. Triplicate cultures were inoculated in 7H9 broth supplemented with 10% OADC and 0.05% Tween 80 at an initial OD_600_ of 0.05. Cultures were incubated at 37°C with shaking for 72 h. Bacterial growth was monitored by measuring OD_600_ every 6 h, and growth curves were plotted to compare the proliferation rates among the three strains.

### Size-exclusion chromatography

The protocol to express and purify NudC^Mab^ was according to the previous study (24). Briefly, pET-28a vector, *E. coli* BL21 (λDE3), and isopropyl-β-D-thiogalactopyranoside were used to express the NudC^Mab^, and then his-tag protein purification kit (Beyotime) was performed to purify the protein. Size-exclusion chromatography was performed to determine the oligomeric states of purified NudC and NudC^P226Q^ proteins. Protein samples were applied to a HiLoad 16/600 Superdex 75 pg column equilibrated with 20 mM Tris-HCl (pH 7.5), 150  mM NaCl, and 1 mM 1,4-dithiothreitol. Chromatography was carried out at 4°C on an ÄKTA Pure system at a flow rate of 1 mL/min, and elution was monitored at 280 nm. Retention times were compared to molecular weight standards to determine oligomeric states.

### Extraction of ETH metabolites

Putative ETH-NAD adduct was separated according to the previous studies using chromatography method (22, 31). The Wt and ΔnudC were grown to an OD_600_ of 0.7 and then exposed to ETH at 256 µg/mL for an additional 3 h with shaking at 37°C. The bacteria were harvested by centrifugation at 13,000 × *g* for 10 min at 4°C and washed with deionized water twice at the same centrifugation condition. The resulting bacterial pellets were resuspended in 6 mL of chloroform-methanol (2:1) and incubated at room temperature and 200 rpm for 3 h, and the solvents were evaporated using CentriVap (Labconco). The dried bacterial precipitate was resuspended in 100 mM ammonium bicarbonate (pH 7.0) and physically disrupted through vigorous mixing using both 0.1 mm and 0.5 mm zirconia beads. The lysate was centrifuged at 18,000 × *g* for 20 min at 4°C. The supernatant was collected and passed through a 0.22 µM filter (Millipore) to remove macromolecular products. The formic acid was used to acidify the final filtrate to a final concentration of 0.1% and centrifuged at 18,000 × *g* for 20 min at 4°C, and 2 µL of the product was subjected to HPLC/MS (Ultimate3000/Q-Exactive HF-X, ThermoFisher Scientific) analysis.

### ETH-NAD adduct detection

The ETH-NAD adduct and their hydrolysis products were analyzed by HPLC/MS. Samples (2 µL) were injected onto an ACQUITY UPLC BEH C18 (100 × 2.1 mm, 1.7 µm particle size, Waters), and separated using a 0.3 mL/min gradient of acetonitrile with 0.1% formic acid (mobile phase B) and 0.1% formic acid in water (mobile phase A) as follows: 0–4 min: 1%–90% B, 4.01–5 min: 90% B, followed by 2 min re-equilibration at 1% B. MS working parameters were as follows: sheath gas flow rate: 40; aux gas flow rate: 10; spray voltage: 3.5 kV; capillary temperature: 300°C; S-lens RF level: 55; and aux gas heater temperature: 350°C. Fragmentation spectra were generated using a narrow quad isolation window of 1 amu and subject to CEs of 30V. All MS data were acquired in a scan range between 100 and 1,000 *m*/*z* under the positive ionization mode.

### Statistical analysis

The statistical analyzes were performed using GraphPad Prism 10.1.2 (GraphPad software). The data comparison between the two experimental groups was analyzed using an independent sample *t*-test. When *P* < 0.05, the difference in experimental results is statistically significant.

## SUPPLEMENTAL MATERIAL

Supplemental material spectrum.02889-25-s0001.pdf Fig. S1 to S6.

ASM does not own the copyrights to Supplemental Material that may be linked to, or accessed through, an article. The authors have granted ASM a non-exclusive, world-wide license to publish the Supplemental Material files. Please contact the corresponding author directly for reuse.
