# Supplementary material for: The MAB_3513c gene plays a key role in intrinsic resistance of Mycobacterium abscessus to isoniazid and ethionamide
Source: Microbiol Spectr. 2025 Nov 28;14(1):e02889-25. doi: 10.1128/spectrum.02889-25 (PMC12772312; doi:10.1128/spectrum.02889-25)
Supplement: Supplemental material — Fig. S1 to S6. [file spectrum.02889-25-s0001.pdf]

The *MAB_3513c* gene plays a key role in intrinsic resistance of *Mycobacterium abscessus* to isoniazid and ethionamide

Shuai Wang [a, b, c#], Xiaofan Zhang [a, b, c, d#], Xiange Fang [a, b, c#], H.M. Adnan Hameed [a, b, c], Abdul Malik [a, b, c], Lihua Long [a, b, c], Yamin Gao [a, b, c], Cuiting Fang [a, c, f, g], Xirong Tian [a, c, e, f], Jinxing Hu [g], Xingyue Wang [e, g], Liqiang Feng [a, b, c], Tianyu Zhang [a, b, c, e, f, g*]

[a] State Key Laboratory of Respiratory Disease, Guangzhou Institutes of Biomedicine and Health, Chinese Academy of Sciences, Guangzhou 510530, China

[b] University of Chinese Academy of Sciences, Beijing 100049, China

[c] China-New Zealand Joint Laboratory on Biomedicine and Health, Guangzhou Institutes of Biomedicine and Health, Chinese Academy of Sciences, Guangzhou 510530, China

[d] Department of Clinical Laboratory, Sun Yat-sen Memorial Hospital of Sun Yat-sen University, Guangzhou 510120, China

[e] Guangzhou National Laboratory, Guangzhou 510320, China

[f] State Key Laboratory of Respiratory Disease, Guangzhou Medical University, Guangzhou 510230, China

[g] State Key Laboratory of Respiratory Disease, Guangzhou Chest Hospital, Guangzhou 510095, China

[*] Correspondence: Tianyu Zhang, zhang_tianyu@gibh.ac.cn

# These authors contributed equally to this work, and the order of authorship was determined by seniority.

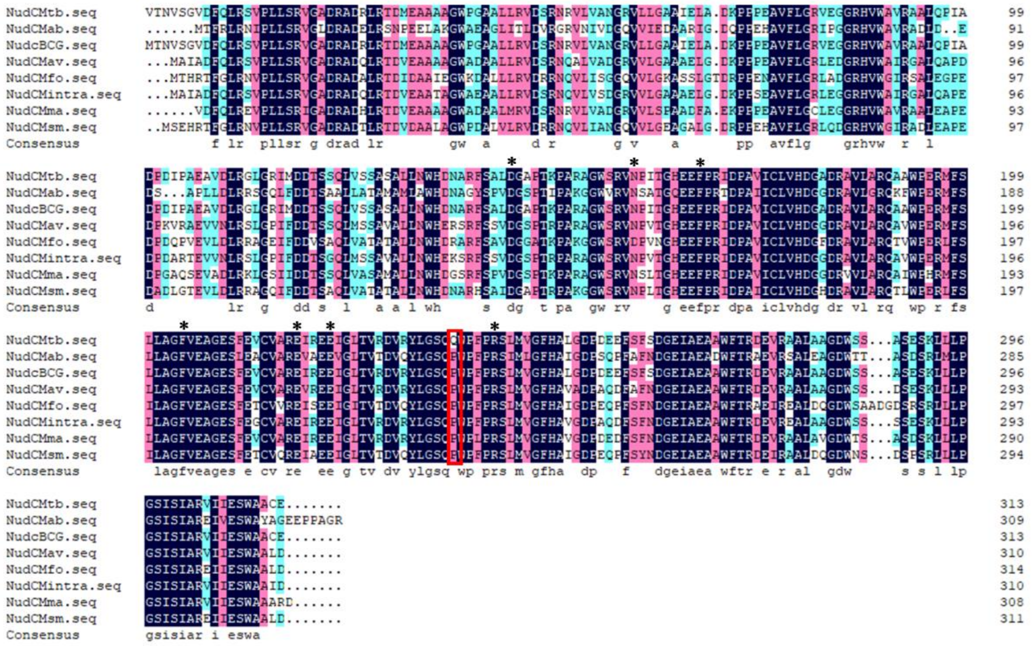

Fig. S1 Alignment of the multiple NudC amino acid sequences from mycobacteria. The NudC at position 226 (*M. abscessus* numbering) is represented in a red square. The other seven key amino acid active sites (D133, N148, F156, F193, E207, E211, and R231) were marked with asterisk.

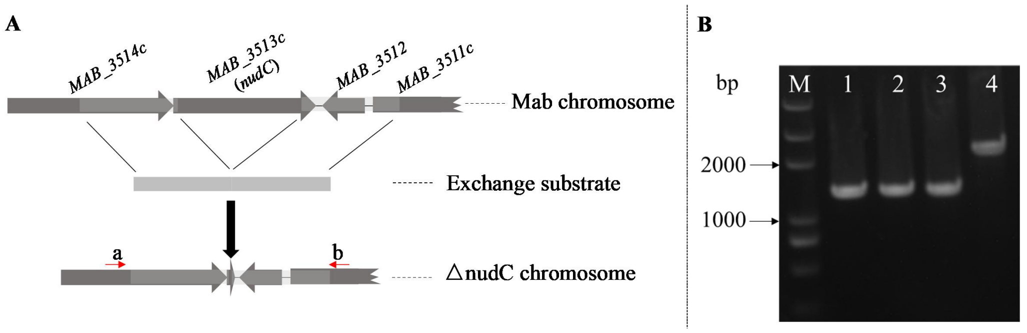

Fig. S2 Construction of a selectable marker-free *nudC* deletion mutant in *M. abscessus*. (A) Schematic representation of the strategy used to generate a selectable marker-free deletion of *nudC*. (B) PCR verification of the deletion. Lane M: DNA ladder; lanes 1-3: PCR products from independent ΔnudC mutants using primers a and b; lane 4: PCR product from wild-type *M. abscessus* using the same primers. The expected band sizes are ~2.4 kb for the wild-type allele and ~1.5 kb for the deletion allele.

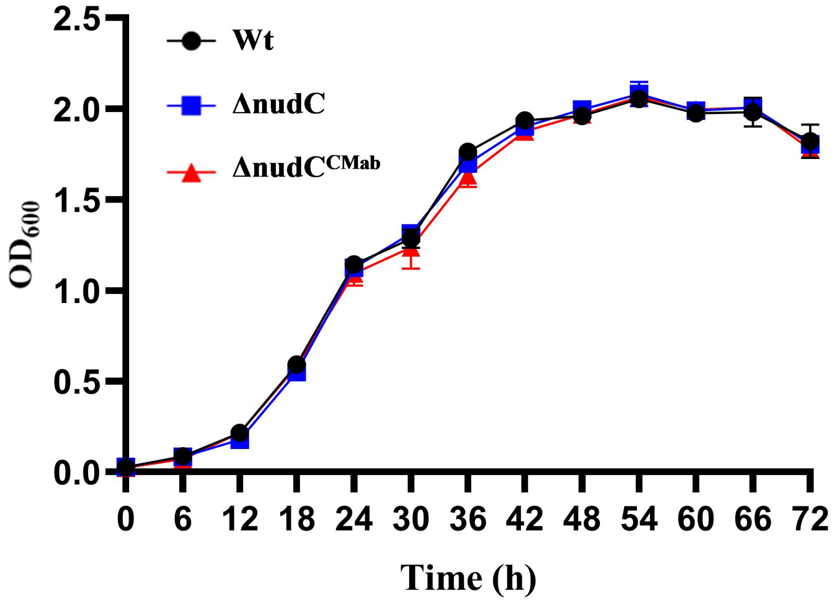

Fig. S3 Growth curves of the *M. abscessus*, ΔnudC and ΔnudC^{CMab} strains in 7H9 broth at 37°C.

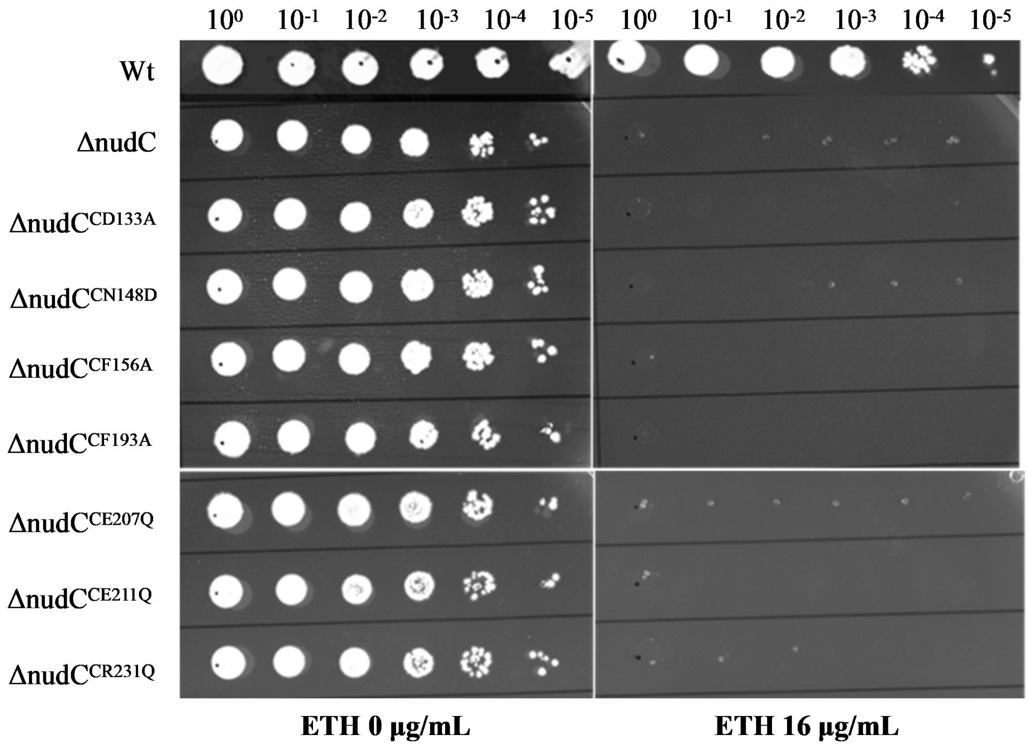

Fig. S4 The sensitivity of ΔnudC strain and the strains complemented with a *nudC* encoding a point mutation (D133A, N148D, F156A, F193A, E207Q, E211Q, or R231Q) to ETH on the 7H11 plates.

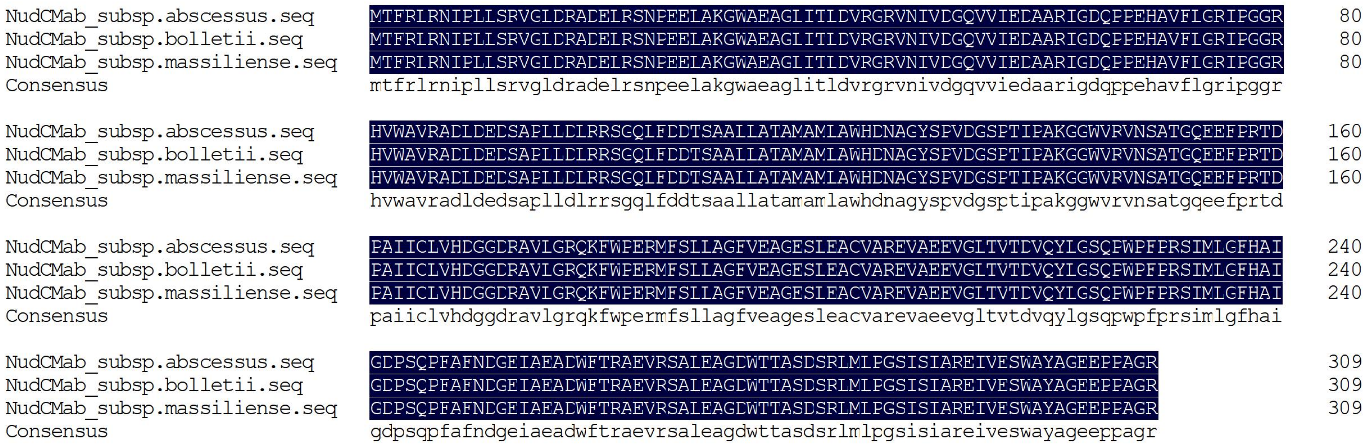

Fig. S5 Alignment of the NudC amino acid sequences from three *M. abscessus* subspecies.

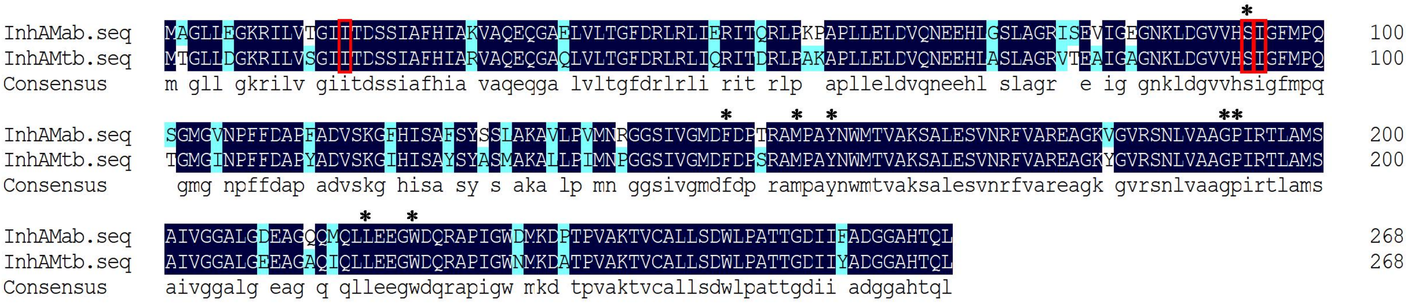

Fig. S6 Alignment of the InhA amino acid sequences from *M. abscessus* and *M. tuberculosis*. Common mutations (S94A, I21T and I95P) of InhAs identified in INH- and ETH-resistant *M. tuberculosis* clinical isolates are represented in a red square. The active sites (S94, F149, M155, Y158, G192, P193, L218 and W222) of InhA interacting with INH-NAD adduct are marked with asterisk.
